# Assessment of eating habits and lifestyle during the coronavirus 2019 pandemic in the Middle East and North Africa region: a cross-sectional study

**DOI:** 10.1017/S0007114520004547

**Published:** 2020-11-17

**Authors:** Leila Cheikh Ismail, Tareq M. Osaili, Maysm N. Mohamad, Amina Al Marzouqi, Amjad H. Jarrar, Antonis Zampelas, Carla Habib-Mourad, Dima Omar Abu Jamous, Habiba I. Ali, Haleama Al Sabbah, Hayder Hasan, Latifa Mohammed Rashid AlMarzooqi, Lily Stojanovska, Mona Hashim, Reyad R. Shaker Obaid, Samar ElFeky, Sheima T. Saleh, Zahieh A. M. Shawar, Ayesha S. Al Dhaheri

**Affiliations:** 1 Clinical Nutrition and Dietetics Department, College of Health Sciences, Research Institute of Medical and Health Sciences (RIMHS), University of Sharjah, Sharjah 27272, United Arab Emirates; 2 Nuffield Department of Women’s & Reproductive Health, University of Oxford, Oxford OX1 2JD, UK; 3 Department of Nutrition and Food Technology, Faculty of Agriculture, Jordan University of Science and Technology, Irbid 22110, Jordan; 4 Department of Nutrition and Health, College of Medicine and Health Sciences, United Arab Emirates University, Al Ain 15551, United Arab Emirates; 5 Department of Food Science and Human Nutrition, Agricultural University of Athens, 11855 Athens, Greece; 6 Nutrition Department, Faculty of Agricultural and Food Sciences, American University of Beirut, Beirut 1107-2020, Lebanon; 7 Research Institute for Medical and Health Sciences, University of Sharjah, Sharjah 27272, United Arab Emirates; 8 Department of Health Sciences, College of Natural and Health Sciences, Zayed University, Dubai 19282, United Arab Emirates; 9 Nutrition Department, Ministry of Health and Prevention, Dubai 1853, United Arab Emirates; 10 Institute for Health and Sport, Victoria University, Melbourne 14428, Australia; 11 Nutrition and Dietetics Program, School of Health Sciences, Universiti Sains Malaysia, Kubang Kerian 16150, Kelantan, Malaysia; 12 Community-Based Initiatives and Health for Older People, Department of Healthier Population, World Health Organization, Eastern Mediterranean Regional Office, Cairo 7608, Egypt; 13 Nutrition Department, Hebron Governmental Hospital, Ministry of Health, Hebron 198, Palestine

**Keywords:** Coronavirus disease 2019, Middle East and North Africa region, Eating habits, Lifestyle behaviours, Dietary patterns

## Abstract

Coronavirus disease 2019 (COVID-19) has rapidly spread globally, forcing countries to apply lockdowns and strict social distancing measures. The aim of this study was to assess eating habits and lifestyle behaviours among residents of the Middle East and North Africa (MENA) region during the lockdown. A cross-sectional study among adult residents of the MENA region was conducted using an online questionnaire designed on Google Forms during April 2020. A total of 2970 participants from eighteen countries participated in the present study. During the pandemic, over 30 % reported weight gain, 6·2 % consumed five or more meals per d compared with 2·2 % before the pandemic (*P* < 0·001) and 48·8 % did not consume fruits on a daily basis. Moreover, 39·1 % did not engage in physical activity, and over 35 % spent more than 5 h/d on screens. A significant association between the frequency of training during the pandemic and the reported change in weight was found (*P* < 0·001). A significantly higher percentage of participants reported physical and emotional exhaustion, irritability and tension either all the time or a large part of the time during the pandemic (*P* < 0·001). Although a high percentage of participants reported sleeping more hours per night during the pandemic, 63 % had sleep disturbances. The study highlights that the lockdown due to the COVID-19 pandemic caused a variety of lifestyle changes, physical inactivity and psychological problems among adults in the MENA region.

The outbreak of the 2019 novel coronavirus disease (COVID-19) was first reported in late December 2019 solely in the city of Wuhan, China^(1)^. Despite strategies adopted by the Chinese government to stop the infection, it continued to spread throughout the world. By the end of January 2020, WHO declared COVID-19 as a public health emergency of international concern^(2)^ and on 11 March 2020, WHO characterised this epidemiological phenomenon as a global pandemic^(3)^. According to the situation report published by the WHO on 5 July 2020, there were over 11 million confirmed cases globally and about 1·1 million cases in the Eastern Mediterranean Region^(4)^. In the Middle East and North Africa (MENA) region, the Gulf countries like Saudi Arabia, Qatar, United Arab Emirates and Kuwait reported the highest numbers of confirmed cases proportionally to the population size^(4)^. According to the Organization for Economic Cooperation and Development, some countries in the MENA region have taken crucial measures to combat this pandemic, closing schools, kindergartens, religious places, airports and malls, as well as preventing social gatherings. Others have gone far by suspending government departments^(5)^.

With more knowledge acquired on the virus, its rate of transmission, resemblance to previous outbreaks and the uncontrolled spread of the virus, governments worldwide had to act quickly^(6)^. Following the footsteps of China and the recommendations of the WHO, governments around the globe started implementing isolation, quarantine, social distancing and community containment at different magnitudes in order to reduce the spread of the virus and lessen its impact on medical resources^(2,7)^.

In most countries, quarantine and physical distancing were initiated, school closures were mandatory and large gatherings were cancelled^(8)^. People were forced to stay at home, practice remote working and online learning from home and adhere with physical distancing^(9)^. However, initiating such sudden changes proposes major modifications in the lifestyle and behaviours of the population. Personal restrictions might lead to physical inactivity especially in courtiers with complete lockdowns, like Jordan^(10)^. Emotional stress and irritability are common psychological impacts during such times of crisis^(11)^. A recent review on the psychological impact of quarantine reported a negative effect, and the stressor factors included long quarantine period, fear of infection, frustration, boredom, financial loss, stigma, inadequate supplies of goods and misinformation^(12)^. Consequently, boredom and stress have been associated with overeating and greater energy intake mostly from comfort foods which are usually high in fat, sugar, salt and energy content^(13,14)^. Quarantine-related stress might also cause sleep disturbances, which in turn has been linked to increased food intake and weight gain^(15)^.

To date, there is no specific cure or anti-viral medicine to treat COVID-19; therefore, maintaining healthy nutritional habits, following food safety recommendations, regular physical activity, coping with stress and adequate sleeping hours are of utmost importance during quarantine^(16)^.

Despite the global administration of quarantine, little is known about its effect on the daily life of the general population in the MENA region. Therefore, the objective of this study was to assess eating habits and other lifestyle behaviours among residents of the MENA region after implementing lockdown and travel restrictions. Moreover, understanding the effect quarantine has on eating habits and lifestyle on individuals could be proved essential in designing intervention protocols in the possibility of another lockdown caused by a new wave of the same or mutated virulent virus.

## Methods

### Study design and participants

This cross-sectional, online survey was conducted in the Greater Middle East region between 15 April 2020 and 29 April 2020. The sample was drawn from eighteen countries within the MENA region, including Algeria, Bahrain, Egypt, Iraq, Jordan, Kuwait, Lebanon, Libya, Morocco, Oman, Palestine, Qatar, Saudi Arabia, Sudan, Syria, Tunisia, United Arab Emirates and Yemen. Adults aged 18 years and older were recruited in the study using a convenience and snowball sampling method. This method depends on data collection from population members who are conveniently available to participate in the study. There was no restriction on the total number of participants; however, we aimed at a minimum of 100 participants from each country.

### Survey questionnaire

A multicomponent, self-administrated online questionnaire was designed using Google Forms in English, Arabic and French. Two researchers from the College of Health Sciences at the University of Sharjah (United Arab Emirates) and the College of Food and Agriculture at United Arab Emirates University (United Arab Emirates) developed the initial draft of the questionnaire in English. The questions were developed based on the Short FFQ^(17)^, the International Physical Activity Questionnaire Short Form^(18)^ and the Copenhagen Psychosocial Questionnaire^(19)^. The questions were then translated to Arabic and French and culturally adapted following internationally accepted methodology^(20,21)^. The questionnaire was then reviewed by other members of the research team and was pilot-tested with thirty people from five countries in the MENA region. Minor modifications in wording were made to the questionnaire following the pilot-testing. The questionnaire that included fifty-eight questions was divided into seven sections: (1) socio-demographic background (thirteen items): age, sex, country of residence, education level, employment status, marital status, number of children, height, weight, health status, a work or study setting; (2) sources of information (two items): source of health-related information and source of nutrition-related information; (3) eating habits (nineteen items): meal type, meal frequency, eating breakfast, skipping meals, water intake, food choices, changes in food intake, following a diet, taking supplements and consuming herbs; (4) shopping habits (six items): using a grocery list, stocking up, online shopping, reading food labels and cleaning/sanitising groceries; (5) physical activity (eight items): exercising frequency and duration, household chores frequency and duration and sedentary time for work, study and entertainment; (6) stress and irritability (two items): physical exhaustion, emotional exhaustion, irritability and tension and (7) sleep (eight items) sleep duration, sleep quality, sleep disturbances and energy level. The full version of the questionnaire is available in online Supplementary Appendix 1.

The survey was distributed through emails and social media platforms. An information sheet and consent form appeared on the first page of the survey indicating the study description, main objective and the right of participants to withdraw at any time. The consenting participants then chose their desired language of communication and proceeded to complete and submit the questionnaire. All data were collected anonymously, and no incentives were given for completing the questionnaire.

This study was conducted according to the guidelines laid down in the Declaration of Helsinki, and all procedures involving human subjects were approved by the Research Ethics Committee at the University of Sharjah (REC-20-04-25-02) and the Social Sciences Research Ethics Committee at the United Arab Emirates University (ERS_2020_6106). Electronic informed consent was obtained from all participants.

### Statistical analysis

All variables presented in this study are of categorical nature since they represent population characteristics. Categorical variables are presented as frequencies and percentages (%; relative frequency × 100). The *χ* ^2^ test was used to examine group differences for single observations in categorical variables, and McNemar’s test was used to investigate the difference between categorical variables before and during the COVID-19 pandemic (accounting for dependent observations). Results were significant for *P* value < 0·05. Statistical analysis was performed using Statistical Package for the Social Sciences version 26.0 (IBM).

## Results

### Demographic characteristics

The questionnaire was completed by 2970 participants. Most of them completed the survey in Arabic language (63·1 %) followed by English (33·8 %) and French (3 %). The demographic characteristics of the study population are presented in Table 1.

**Table 1.** Demographic breakdown of surveyed participants (*n* 2970) (Frequencies and percentages)

	*n*	%
Sex		
Male	844	28·4
Female	2126	71·6
Age (years)		
18–25	879	29·6
26–35	784	26·4
36–45	668	22·5
46–55	443	14·9
>55	196	6·6
Marital status		
Married	1579	53·2
Single	1264	42·6
Divorced	94	3·2
Widowed	33	1·1
Number of children		
None	1478	49·7
1–2	753	25·4
≥3	739	24·9
Education level		
Less than high school	40	1·3
High school	239	8·0
College/diploma	457	15·4
Bachelor’s degree	1439	48·5
Higher than bachelor’s degree	795	26·8
Employment status		
Full time	1331	44·8
Part time	177	6·0
Self-employed	226	7·9
Student	572	19·3
Unemployed	563	19·0
Retired	101	3·4
Working/studying from home		
Yes	1663	56·0
No	1109	37·3
Not applicable	198	6·7
Weight change during pandemic		
Lost weight	503	16·9
Gained weight	901	30·3
Maintained weight	1305	43·9
Do not know	261	8·8
Health state during pandemic		
Excellent	623	21·0
Very good	1100	37·0
Good	931	31·3
Fair	290	9·8
Poor	26	0·9
Country of residence		
United Arab Emirates	390	13·1
Jordan	353	11·9
Lebanon	343	11·5
Saudi Arabia	246	8·3
Palestine	189	6·4
Bahrain	171	5·8
Sudan	166	5·6
Algeria	159	5·4
Iraq	145	4·9
Syria	138	4·6
Kuwait	135	4·5
Egypt	134	4·5
Oman	78	2·6
Tunisia	77	2·6
Yemen	73	2·5
Libya	69	2·3
Qatar	54	1·8
Morocco	50	1·7

The male:female ratio was almost 1:3, with 28·4 % males. The majority of surveyed individuals were aged 18–25 years (29·6 %), were married (53·2 %), had no children (49·7 %), completed a university degree (48·5 %), worked full-time (44·8 %) and were working/studying from home during the lockdown (56·0 %). About one-third of the surveyed individuals reported weight gain during the COVID-19 pandemic (30·3 %), 16·9 % lost weight, 43·9 % maintained weight and 8·8 % did not know if there was a change in their weights. When asked to describe the general state of their health during the outbreak, the majority of surveyed individuals reported very good health state (37·0 %) and only 0·9 % reported poor state of health.

Eighteen countries from the MENA region (Algeria, Bahrain, Egypt, Iraq, Jordan, Kuwait, Lebanon, Libya, Morocco, Oman, Palestine, Qatar, Saudi Arabia, Sudan, Syria, Tunisia, United Arab Emirates and Yemen) participated in the survey. The largest proportion of respondents was from the United Arab Emirates (13·1 %), Jordan (11·9 %), Lebanon (11·5 %), Saudi Arabia (8·3 %) and Palestine (6·4 %).

### Sources of information

Table 2 presents sources of health- and nutrition-related information among the surveyed participants. Social media applications were the most common resource of information for both health and nutrition updates (70·3 and 70·8 %, respectively), followed by local and international health authorities for health-related information (53·9) and healthcare professionals for nutrition-related information (41·3 %).

**Table 2.** Sources of health and nutrition information during coronavirus disease 2019 (COVID-19) pandemic (*n* 2970) (Frequencies and percentages)

Sources of information*	Health-related information		Nutrition-related information	
	*n*	%	*n*	%
Local and international health authorities	1602	53·9	1192	40·1
Social media	2089	70·3	2104	70·8
Healthcare professionals	1145	38·6	1228	41·3
Television	887	29·9	654	22·0
Newspapers	162	5·5	110	3·7
Friends and family	947	31·9	1047	35·3

* As multiple responses were allowed, the total number of responses is greater than the number of surveyed participants and the percentage of cases is displayed.

### Eating habits

The results on eating habits (Table 3) showed an increase in the number of meals consumed per d during the COVID-19 pandemic compared with before the pandemic. The percentage of participants consuming five or more meals per d increased from 2·2 % before the pandemic to 6·2 % during the pandemic (*P* < 0·001). Moreover, the percentage of participants skipping meals decreased from 64·4 % before the pandemic to 45·1 % during the pandemic (*P* < 0·001). Among those who reported skipping meals, 60·8 % stated that lack of time was the main reason before the pandemic and 37·9 % stated that lack of appetite was the main reason for skipping meals during the pandemic. Although the results showed an increase in meal frequency, 74·0 % of participants reported not meeting the recommended water intake by drinking less than eight cups of water per d during the pandemic.

**Table 3.** Eating habits pre- and during coronavirus disease 2019 (COVID-19) pandemic (*n* 2970) (Frequencies and percentages)

	Pre-COVID-19		During COVID-19		*P* (2-sided)
	*n*	%	*n*	%	
Most consumed meals during the week*					
Home-made	2517	84·7	2886	97·2	<0·001
Frozen ready-to-eat meals	249	8·4	222	7·5	0·094
Fast food	856	28·8	158	5·3	<0·001
Restaurants	686	23·1	123	4·1	<0·001
Healthy restaurants	284	9·6	90	3·0	<0·001
Number of meals per d					
1–2 meals	1355	45·6	1115	37·5	<0·001
3–4 meals	1549	52·2	1670	56·2	<0·001
≥5 meals	66	2·2	185	6·2	<0·001
Eating breakfast on most days					
Yes	1986	66·9	2114	71·2	<0·001
No	984	33·1	856	28·8	
Skipping meals					
Yes	1912	64·4	1340	45·1	<0·001
No	1058	35·6	1630	54·9	
Reasons for skipping meals (if the answer was yes)*					
To reduce food intake	353	18·6	367	27·7	<0·001
Lack of time	1153	60·8	357	27·0	<0·001
To lose weight	382	20·2	315	23·8	0·004
Lack of appetite	586	30·9	502	37·9	0·472
Fasting	189	10·0	349	26·4	<0·001
Amount of water consumed per d					
1–4 cups	1225	41·2	1042	35·1	<0·001
5–7 cups	1069	36·0	1155	38·9	0·002
≥8 cups	676	22·8	773	26·0	<0·001

* As multiple responses were allowed, the total number of responses is greater than the number of surveyed participants and the percent of cases is displayed. *P* values based on *α* = 0·05 level of significance following McNemar’s test.

Table 4 presents the frequency of consumption for particular food products during the COVID-19 pandemic among MENA participants. As shown, 48·8 % of surveyed participants did not consume fruits on a daily basis and 32·5 % did not consume vegetables daily. However, 44·1 % of participants reported consuming sweets or desserts at least once every day and 32·9 % consumed salty snacks (chips, crackers and nuts) daily. Moreover, 70·5 % of participants had tea or coffee at least once per d. Energy drinks were less popular among the study participants compared with sweetened drinks, as 22·5 % of those surveyed reported consuming sweetened drinks at least once per d and only 4·5 % consumed energy drinks daily.

**Table 4.** Frequency of consumption of particular foods during coronavirus disease 2019 (COVID-19) pandemic (*n* 2970) (Frequencies and percentages)

Food items	≥4 times/d		2–3 times/d		Once/d		1–4 times/week		Never	
	*n*	%	*n*	%	*n*	%	*n*	%	*n*	%
Fruits	47	1·6	430	14·5	1044	35·2	1292	43·5	157	5·3
Vegetables	113	3·8	740	24·9	1152	38·8	908	30·6	57	1·9
Milk and milk products	50	1·7	484	16·3	1141	38·4	1040	35·0	255	8·6
Meat/fish/chicken	51	1·7	346	11·6	1225	41·2	1274	42·9	74	2·5
Bread/rice/pasta	127	4·3	818	27·5	1030	34·7	899	30·3	96	3·2
Sweets/desserts	78	2·6	339	11·4	894	30·1	1328	44·7	331	11·1
Salty snacks	38	1·3	201	6·8	737	24·8	1473	49·6	521	17·5
Coffee/tea	235	7·9	933	31·4	927	31·2	645	21·7	230	7·7
Sweetened drinks (soda, juice)	44	1·5	174	5·9	448	15·1	983	33·1	1321	44·5
Energy drinks	9	0·3	21	0·7	103	3·5	286	9·6	2551	85·9

### Shopping

More than two-thirds of the respondents (65·7 %) sanitised their groceries before storage. Moreover, 76·4 % of participants reported preparing a list before grocery shopping. Online shopping was less common among the surveyed participants as only one-third reported ordering groceries online (Table 5).

**Table 5.** Shopping practices during coronavirus disease 2019 (COVID-19) pandemic (*n* 2970) (Frequencies and percentages)

	*n*	%
Prepare shopping list		
Yes	2268	76·4
No	702	23·6
Started stocking up on foods		
Yes	1370	46·1
No	1023	34·4
Already stocking up	577	19·4
Ordering groceries online		
Yes	1053	35·5
No	1917	64·5
Reading food labels		
Yes	1573	53·0
No	278	9·4
Sometimes	1119	37·7
Sanitising/cleaning groceries		
Yes	1952	65·7
No	419	14·1
Sometimes	599	20·2

**Table 6.** Daily activities pre- and during coronavirus disease 2019 (COVID-19) pandemic (*n* 2970) (Frequencies and percentages)

	Pre-COVID-19		During COVID-19		*P* (2-sided)*
	*n*	%	*n*	%	
Doing household chores					
Never	855	28·8	586	19·7	<0·001
1–3 times/week	988	33·3	800	26·9	<0·001
4–5 times/week	180	6·1	295	9·9	<0·001
Every day	947	31·9	1289	43·4	<0·001
Screen time for study or work					
None	663	22·3	693	23·3	0·105
1–2 h/d	913	30·7	595	20·0	<0·001
3–5 h/d	680	22·9	656	22·1	0·438
>5 h/d	714	24·0	1026	34·5	<0·001
Screen time for entertainment					
<30 min/d	354	11·9	149	5·0	<0·001
1–2 h/d	1310	44·1	642	21·6	<0·001
3–5 h/d	872	29·4	1066	35·9	<0·001
>5 h/d	434	14·6	1113	37·5	<0·001

* *P* values based on *α* = 0·05 level of significance following McNemar’s test.

**Table 7.** Sleep pre- and during coronavirus disease 2019 (COVID-19) pandemic (*n* 2970) (Frequencies and percentages)

	Pre-COVID-19		During COVID-19		*P* (2-sided)
	*n*	%	*n*	%	
Hours of sleep per night					
<7 h	1528	51·4	1086	36·6	<0·001
7–9 h	1346	45·3	1472	49·6	<0·001
>9 h	96	3·2	412	13·9	<0·001
How would you rate your sleep quality					
Very good	844	28·4	758	25·5	<0·001
Good	1617	54·4	1346	45·3	<0·001
Poor	509	17·1	866	29·2	<0·001
Did you experience any of the following*					
Slept badly and restlessly	738	24·8	853	28·7	<0·001
Hard to go to sleep	639	21·5	1035	34·8	<0·001
Woken up too early and not been able to get back to sleep	700	23·6	638	21·5	0·018
Woken up several times and found it difficult to get back to sleep	568	19·1	760	25·6	<0·001
None	1392	46·9	1092	36·8	<0·001
Describe your energy level					
Energised	1100	37·0	581	19·6	<0·001
Neutral	1730	58·2	1509	50·8	<0·001
Lazy	140	4·7	880	29·6	<0·001

* As multiple responses were allowed, the total number of responses is greater than the number of surveyed participants and the percent of cases is displayed. *P* values based on *α* = 0·05 level of significance following McNemar’s test.

### Physical activity

**Fig. 1.**
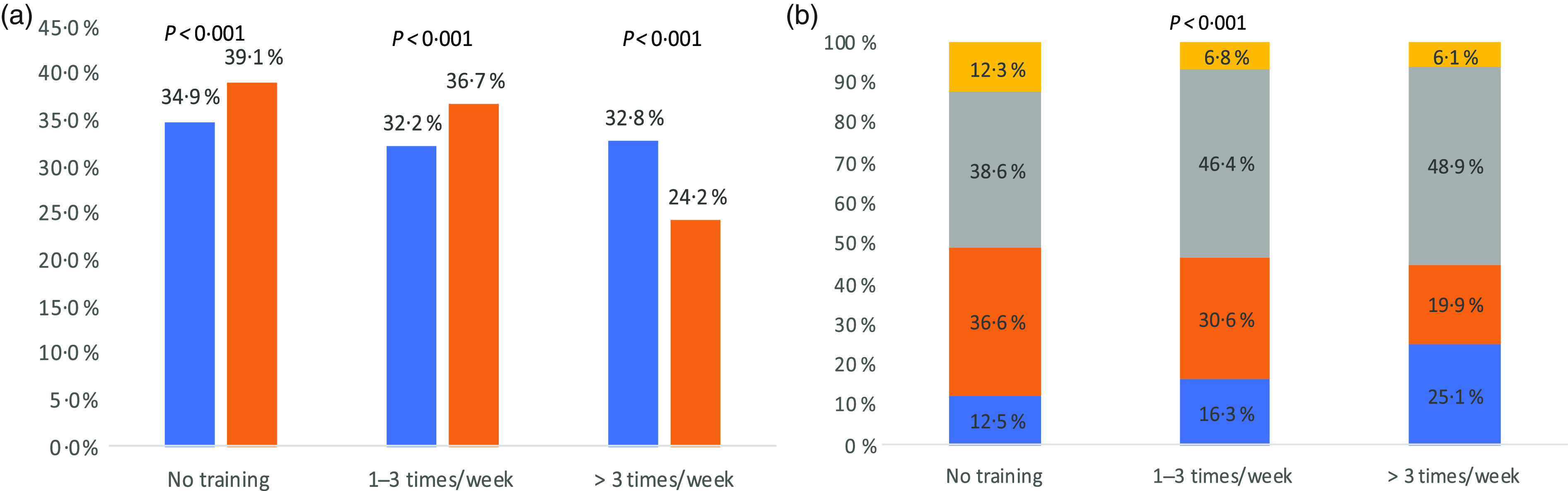
(a) Physical activity pre- and during coronavirus disease 2019 (COVID-19) pandemic (*P* values represent significance levels of McNemar’s test). 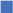, Pre-COVID-19; 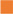, during COVID-19. (b) Physical activity during COVID-19 pandemic and change in weight (*P* values represent significance levels of *χ* ^2^ test). 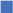, Lost weight; 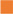, gained weight; 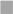, maintained weight; 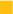, I do not know.

Data about frequency of physical activity among surveyed population are presented in Fig. 1. Over one-third of participants reported not engaging in any physical activity before the COVID-19 pandemic (34·9 %), and even a higher percentage reported not participating in any physical activity during the pandemic (39·1 %) (*P* < 0·001). There was a significant association between the frequency of training during the pandemic and the reported change in weight (*P* < 0·001). Of those who reported training more than three times per week, 25·1 % lost weight and 48·9 % maintained their weight (*P* < 0·001). However, 36·6 % of people who did not train reported gaining weight.

About 35 % of participants spent more than 5 h/d on the computer for study or work purposes during the pandemic, compared with 24·0 % before the COVID-19 lockdown (*P* < 0·001). Moreover, the percentage of participants spending more than 5 h/d on screens for entertainment increased from 14·6 % before the pandemic to 37·5 % during the pandemic (*P* < 0·001) (Table 6).

### Stress

**Fig. 2.**
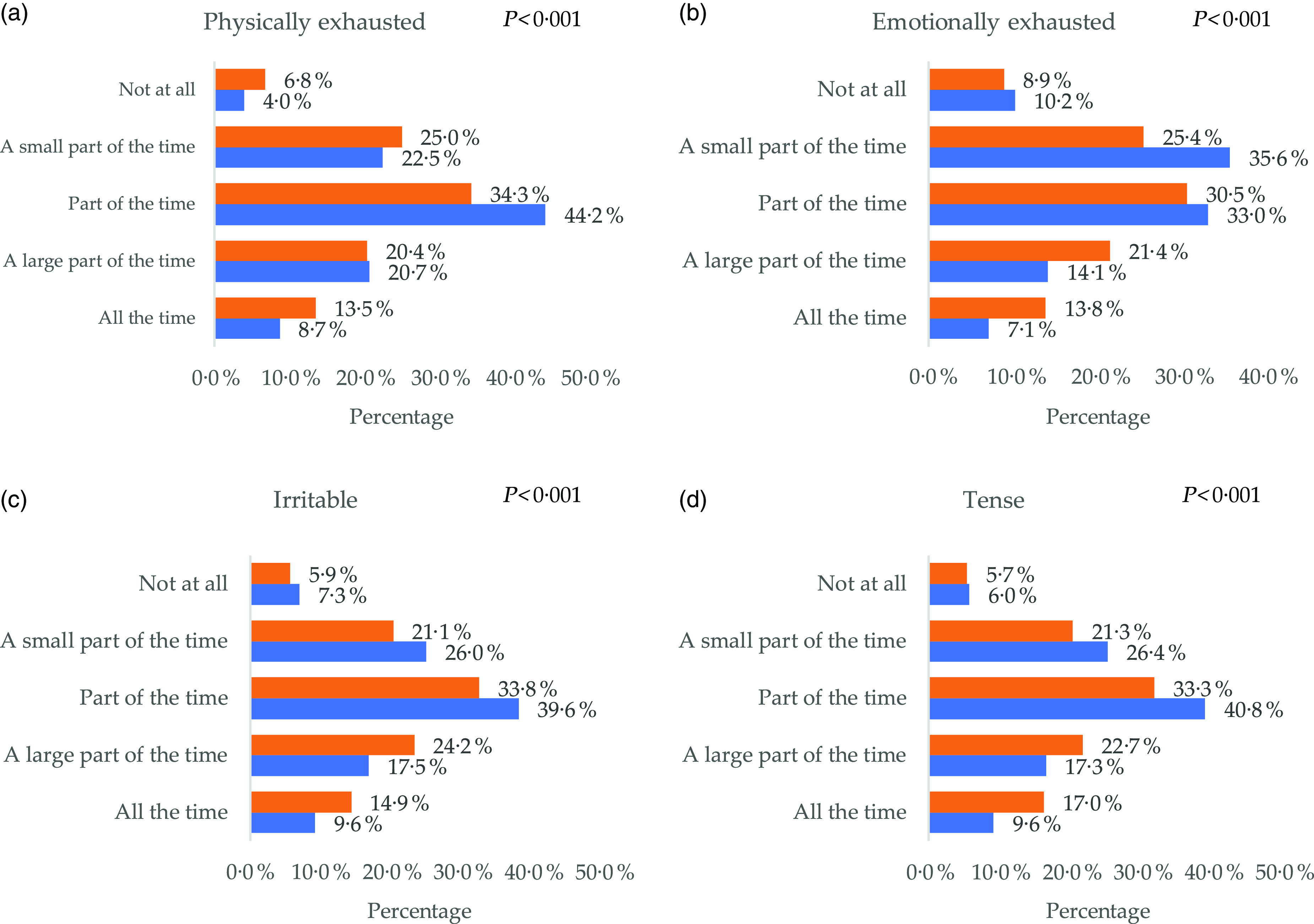
(a) Percentage of participants feeling physically exhausted pre- and during coronavirus disease 2019 (COVID-19) pandemic; (b) percentage of participants feeling emotionally exhausted pre- and during COVID-19 pandemic; (c) percentage of participants feeling irritable pre- and during COVID-19 pandemic and (d) percentage of participants feeling tense pre- and during COVID-19 pandemic (*P* values represent significance levels of McNemar’s test). 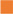, During COVID-19; 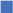, pre-COVID-19.

The results on stress indicate an increase in the percentage of participants reporting physical and emotional exhaustion, irritability and tension all the time or a large part of the time during the COVID-19 pandemic (*P* < 0·001) (Fig. 2).

### Sleep

Although the percentage of participants who were sleeping <7 h per night decreased from 51·4 % before the pandemic to 36·6 % during the pandemic, the percentages of participants reporting poor sleep quality increased from 17·1 % before the pandemic to 29·2 % during the pandemic (*P* < 0·001). Moreover, a higher percentage of participants reported sleep disturbances during the pandemic (63·2 %) compared with before (53·1 %). As a result, 29·6 % of the participants reported feeling lazy and less energised during the pandemic, compared with only 4·7 % before the pandemic (*P* < 0·001) (Table 7).

## Discussion

This cross-sectional study provides a snapshot of the eating habits and lifestyle factors for a sample of 2970 MENA adult residents, who answered an online survey in April 2020, approximately 7–8 weeks following the lockdown implementation to control the spread of the COVID-19 pandemic.

The results of this study showed that about two-thirds of the participants obtained their health- and nutrition-related information mainly through social media applications followed by credible websites of national and international health organisations and healthcare professionals. Similarly, a study in Iran reported that 55·3 % of surveyed nurses obtained their COVID-19 updates and information from the website of the WHO and the Ministry of Health, followed by 48·2 % who relied on social media applications as a main source of information^(22)^. During the COVID-19 outbreak, misinformation overload (‘infodemic’) has bombarded social media, which might have harmed the mental health of individuals^(23)^. A study has shown that 82·0 % of participants were regularly exposed to social media, and the increase of exposure frequency was found associated with higher odds of anxiety and depression^(24)^. Thus, public awareness should be raised about reliable sources of information, especially during such crises.

An increase in the number of meals consumed per d and a reduction in the percentage of skipping meals during the COVID-19 pandemic were reported by the participants in the present study. This also explains the reported weight gain among this population. Likewise, a survey among French adults cohort revealed unfavourable nutritional behaviours of weight gain, decreased physical activity, increased sedentary time, increased snacking, decreased consumption of fresh fruits and vegetables and increased consumption of sweets, biscuits and cakes^(25)^.

About three-quarters of the participants in this study reported drinking less than eight cups of water per d during the COVID-19 pandemic, out of which about a third reported drinking <1 litre/d. These quantities do not meet the recommended water intake set by the WHO of 2·9 litres/d for males and 2·2 litres/d for females^(26)^. Likewise, in Italy, 86·6 % of surveyed participants reported drinking <2 litres of water per d during the COVID-19 pandemic and 26·2 % consumed <1 litre of water per d^(27)^.

Nearly half of the surveyed participants in this study did not consume fruits daily and one-third did not consume vegetables daily. On the other hand, one-third of the same population reported consuming sweets and salty snacks at least once per d. This unfavourable trend towards a Westernised diet was reported in an ecological study in the MENA region (1961–2007), as the proportion of energy derived from meat and vegetable oils increased significantly, while that from cereals, vegetables, fruits, milk and dairy products showed a descending trend^(28)^.

The results clearly demonstrate the need for dietary support of individuals during lockdowns focusing on healthy eating choices. Consuming a diet rich in vegetables and fruits is especially important during these times due to their high content of antioxidants, phytonutrients and anti-inflammatory substances^(29,30)^. A recent meta-analysis of observational studies suggested that consuming fruits and vegetables is negatively associated with the metabolic syndrome and its risk factors^(31)^. In addition to being a rich source of fibre, as well as various micronutrients and antioxidants, an adequate intake of fruits and vegetables might optimise the immunocompetence, a role indicated in both the prevention and treatment of COVID-19^(32)^.

Improving hygiene practices, cleaning groceries before storage and limiting grocery visits have been recommended by various health authorities globally during the COVID-19 pandemic^(33)^. Fortunately, only 14·1 % of study participants did not clean groceries before storage. Likewise, a study in India reported an increase in the frequency of using sanitisers, hand washing and wearing masks among the study participants^(34)^. These findings indicate the increasing awareness of participants towards personal hygienic measures to avoid COVID-19 infection.

In this study, more than one-third of the participants reported not engaging in any physical activity during the COVID-19 pandemic and of those, over one-third reported gaining weight. Recent studies have shown an association between COVID-19 severity and mortality with increased weight and BMI categories among other co-morbidities^(35,36)^. These findings further explain the weight gain witnessed in this study. Obesity rates in the MENA region are already alarmingly high and are associated with non-communicable diseases such as type 2 diabetes, CHD and stroke^(37)^. The unprecedented self-quarantine mandate during the COVID-19 pandemic might have worsened this health problem not only among adults but also among children^(38,39)^. Studies have indicated that physical inactivity results in changes to body composition (e.g. increase in total body fat and abdominal fat) associated with increased insulin resistance, reduced cardiorespiratory fitness and increased dyslipidaemia^(40,41)^. These changes can occur in as little as 2 weeks of physical inactivity and are reversible in younger people, but less so in older adults^(42)^. A recent review suggested similar metabolic consequences during the COVID-19 confinement due to physical inactivity and overeating^(43)^. The review recommended nutritional interventions and resistance training as potential strategies to prevent such deleterious metabolic effects^(43)^. It is challenging, however, to exercise during lockdown when gyms and parks are closed and people are stressed, depressed and isolated^(44,45)^. Awareness about different types of home exercises (resistance training, yoga, pilates, dancing, balance exercise, etc.) and their benefits on weight status and mental health is essential^(46,47)^.

During the COVID-19 pandemic, people are exposed to a stressful situation for an unknown duration of time. This could disrupt sleep quality, which has a direct effect on the physical functioning during the subsequent day^(48)^. Our study found an increase in sleep hours during the pandemic compared with before the pandemic. Moreover, sleep disturbances have been reported by over 60 % of participants, and about 30 % of them had poor sleep quality during the pandemic. Similarly, a survey in Italy reported an increase in the percentage of participants sleeping 7–9 h per night from 49·9 % before the pandemic to 54·8 % during the pandemic^(27)^. In addition, a survey in China indicated that 18·2 % of participants had poor sleep quality, and the percentage increased to about 24 % among healthcare workers^(49)^.

The COVID-19 pandemic has been associated with increased anxiety and distress, which in turn affects the lifestyle of individuals^(50,51)^. A recent review highlighted four different mechanisms that stress can be linked to overweight and obesity including: (1) cognitive processes such as self-regulation; (2) behavioural effect through inducing overeating, shortening of sleep and decreasing physical activity; (3) physiological changes in the hypothalamic–pituitary–adrenal axis and (4) stimulating the production of biochemical hormones and peptides such as leptin, ghrelin and neuropeptide Y^(52)^. Additionally, the main reason for skipping meals among participants in the present study before the pandemic was due to lack of time; however, during the pandemic, participants skipped meals mainly due to lack of appetite, which is common in the presence of depression^(53)^. The results of our study indicate an increase in the percentage of participants reporting physical and emotional exhaustion, irritability and tension all the time, or a large part of the time during the COVID-19 pandemic.

This study was conducted during April 2020. By that time, all countries included in the survey have already declared the state of emergency due to COVID-19^(54)^. The number of confirmed COVID-19 cases by the end of April widely varied between MENA countries and ranged between 21 000 cases in Saudi Arabia and six cases in Yemen^(55)^. Countries like Saudi Arabia, United Arab Emirates and Qatar had over 10 000 confirmed cases by the end of April. However, Jordan, Palestine, Syria, Yemen, Libya and Sudan had <500 cases^(55)^. The number of deaths also varied. United Arab Emirates, Jordan, Lebanon, Palestine, Bahrain, Sudan, Iraq, Syria, Kuwait, Oman, Tunisia, Yemen, Libya and Qatar had <100 deaths by the end of April, while Morocco, Egypt, Algeria and Saudi Arabia reported over 150 deaths in the same period^(55)^. Although there was a variation in the number of cases and deaths between MENA countries, all countries included in the survey implemented strict quarantine measures between mid/end of March 2020 until June 2020 or even longer^(54)^. Countries like Saudi Arabia, Bahrain, Sudan, Egypt and United Arab Emirate did not start easing restrictions until the end of June, while Yemen announced easing lockdown restrictions in mid-July^(54)^. Restrictions included closure of boarders, closure of non-essential businesses, nightly travel curfew, local movement and travel restrictions, facilitating remote working and online learning, and cancelling prayers to avoid mass gathering events^(54)^. Considering different stages of the pandemic in different MENA countries, the survey might not reflect such as diverse behaviour of the entire population from those places.

It is acknowledged that this study has several limitations, including the use of a self-reported questionnaire which might cause some respondent bias or misreporting of data; the cross-sectional study design which does not allow for causality evaluation and the use of convenience sample which limits generalisability of the results; although the aim was to get a minimum of 100 participants from each country, six countries had fewer participants. Also, the information on infection with COVID-19 and medical history was not determined in the study. Such analysis would not only require a longer questionnaire, which might have decreased the compliance and response rate, but also would have required a larger sample size based on the prevalence of all the factors to acquire adequate study power. Another potential limitation of the study might be a recency bias which suggests that recent habits are more easily retrieved from memory; however, data were collected only 1 month after lockdown to minimise memory failure for previous habits. The study information was acquired after lockdown, and although comparisons are critical to be made to draw inferences, no conclusive remarks can be drawn. Nevertheless, developing a web survey allowed a rapid assessment of eating and lifestyle habits in a very critical period of the pandemic and from around eighteen countries in the MENA region. It also facilitated the anonymity of the respondents, thus reducing the social desirability bias. The present study provides unique information about unprecedented circumstances that have faced mankind in recent history.

### Conclusion

The results of the present study suggest that the COVID-19 pandemic triggered a variety of unhealthy lifestyle changes, physical inactivity and psychological problems in the MENA region. There is also substantial need to increase awareness regarding healthy nutritional habits, general safety measures, importance of home-based physical activity and stress relief mechanisms. Finally, the provision of reliable health and nutrition information is essential as well as psychological support during the pandemic.

## Supplementary material

For supplementary material accompanying this paper visit https://doi.org/10.1017/S0007114520004547. click here to view supplementary material
