# Supplementary material for: Assessment of eating habits and lifestyle during the coronavirus 2019 pandemic in the Middle East and North Africa region: a cross-sectional study
Source: Br J Nutr. 2020 Nov 17:1–10. doi: 10.1017/S0007114520004547 (PMC7804075; doi:10.1017/S0007114520004547)
Supplement: Supplementary file 1 [file S0007114520004547sup001.pdf]

Assessment of Eating Habits and Lifestyle during Coronavirus Pandemic in the MENA region: A Cross-Sectional Study

Personal data

1. **What is your gender?**
   - Female
   - Male
2. **Which age group do you belong to?**
   - 18-25
   - 26-35
   - 36-45
   - 46-55
   - >56
3. **Which country do you reside in? (*drop-down of all MENA countries)***
   - _____________
4. **Which city do you reside in?**
   - ______________
5. **What is your marital status?**
   - Married
   - Single
   - Divorced
   - Widowed
6. **How many kids do you have?**
   - Not applicable
   - I have no kids
   - 1-2 kids
   - 3 or more kids
7. **What is your education Level?**
   - Less than high school
   - High School
   - College/ Diploma
   - University Degree
   - Higher education masters/doctorate
   - Other, specify: -----------
8. **What is your employment status?**
   - Full-time employment
   - Part-time employment
   - Unemployed
   - Retired

- Self-employed
- Student

9. Do you currently work/study from home?

- Yes
- No
- Not applicable

10. Did your weight change during coronavirus pandemic?

- Lost weight
- Gained weight
- Maintained weight
- I don't know

11. What is your current weight? __________(kg)

12. What is your height? ___________ (cm)

13. What option describes your general state of health in the past 3 months?

- Excellent
- Very good
- Good
- Fair
- Poor

Sources of information

1. Where do you get health-related information from? ***(choose all that apply)***

- Local and international health authorities
- Websites and social media
- Healthcare professionals
- Television
- Newspapers
- Friends and family

2. Where do you get food and nutrition related information from? ***(choose all that apply)***

- Local and international health authorities
- Websites and social media
- Healthcare professionals
- Television
- Newspapers
- Friends and family

Eating Habits

1. **Please choose an option that best describes your food choices before coronavirus pandemic:** ***(1 = Very healthy and 10 is Extremely unhealthy)***

   1 2 3 4 5 6 7 8 9 10

2. **During coronavirus pandemic, which option best describes your food choices?** ***(1 = Very healthy and 10 is Extremely unhealthy)***

   1 2 3 4 5 6 7 8 9 10

3. **Did your food intake change during coronavirus pandemic?**
   - Yes, it increased
   - Yes, it decreased
   - No, it did not change
4. **Are you following a specific meal plan (diet) during coronavirus pandemic?**
   - Yes, I started a new diet
   - Yes, I was already on a diet
   - No

4.1 If yes, specify what kind of diet you are following

- Low-calorie diet
- Low-fat diet
- Low-carb diet
- Atkins (high protein)
- Ketogenic diet (high fat)
- Vegetarian/ Vegan diet
- Others, ______________

5. **Which of the following factors would motivate you to improve your eating habits? (choose all that apply)**
   - Higher income
   - Less working hours
   - Having health risks
   - Having adequate nutrition information
   - Following an exercise regimen
   - Advice from doctor/dietitian/friend
   - Feeling guilt or shame about body size
6. **Before coronavirus pandemic, most of your consumed meals during the week were?**
   - Homemade
   - Frozen ready-to-eat meals
   - Fast food (take-away, delivery)

- Restaurants (take-away, delivery)
- Healthy food (take-away, delivery)

7. **During coronavirus pandemic most of your consumed meals during the week are?**
    - Homemade
    - Frozen ready-to-eat meals
    - Fast food (take-away, delivery)
    - Restaurants (take-away, delivery)
    - Healthy food (take-away, delivery)
8. **How many meals did you eat per day before coronavirus pandemic?**
    - 1-2
    - 3-4
    - More than 5
9. **How many meals do you eat per day during coronavirus pandemic?**
    - 1-2
    - 3-4
    - More than 5
10. **Did you use to eat breakfast on most days of the week before coronavirus pandemic?**
    - Yes
    - No
11. **During coronavirus pandemic, do you eat breakfast on most days of the week?**
    - Yes
    - No
12. **Before coronavirus pandemic, did you use to skip meals?**
    - Yes
    - No

12.1 If yes, why is that?

- To reduce food intake
- Lack of time
- To lose weight
- Lack of appetite
- Fasting

13. **During coronavirus pandemic, are you skipping meals?**
    - Yes
    - No

13.1 If yes, why is that?

- To reduce food intake

- Lack of time
- To lose weight
- Lack of appetite
- Fasting

14. During coronavirus pandemic, did you start taking supplements (multivitamin, vitamin C, etc.)?

- Yes
- No
- Yes, I already take supplements

14.1 If yes, which type of supplements are you taking? (Choose all that apply)

- Multivitamin
- Vitamin C
- Antioxidants
- Vitamin D
- Vitamin B12
- Fish oil supplements
- Probiotics
- Others, _________________

15. During coronavirus pandemic, are you consuming specific herbs or spices?

- Yes
- No
- Yes, I already consume herbs and spices

16. What do you think increases your immunity against disease? ***(choose all that apply)***

- Eating balanced diet
- Taking supplements
- Drinking adequate fluids
- Practicing physical activity
- Consuming herbs and spices
- Getting proper sleep
- Managing/ Minimizing stress
- Others, __________

17. Before coronavirus pandemic, how much water did you use to drink daily?

- 1-4 cups
- 5-7 cups
- 8 cups or more

18. During coronavirus pandemic, how much water do you drink daily?

- 1-4 cups

- 5-7 cups
- 8 cups or more

19. How often do you eat the following foods listed below?

Food Item	Never	1-4 times/week	Once/day	2-3 times/day	4 or more times/day
Fruits					
Vegetables					
Milk and milk products					
Meat/Chicken/Fish					
Bread/rice/pasta					
Sweets/ desserts					
Coffee/Tea					
Sweet drinks (soft drinks, canned juice, etc.)					
Energy drinks					

Shopping

1. **Do you usually prepare a list before grocery shopping?**
   - Yes
   - No
2. **During coronavirus pandemic, did you start stocking up on food?**
   - Yes
   - No
   - I already stock up on food
3. **During coronavirus pandemic, what food items do you stock up on the most? (choose all that apply)**
   - Fresh fruits and vegetables
   - Fresh meats (meat, fish, chicken, etc.)
   - Frozen fruits and vegetables
   - Canned food (beans, corn, legumes, etc.)
   - Dry food (rice, pasta, pulses, bulgur, etc.)
   - Shelf-stable Milk/ powdered milk.
4. **Would you rather order your groceries online (delivered to your house)?**
   - Yes
   - No
5. **Do you check food labels before purchasing?**
   - Yes
   - No
   - Sometimes
6. **Do you clean and sanitize your groceries before storage?**

- Yes
- No
- Sometimes

Physical Activity

1. **Did you use to exercise before coronavirus pandemic?**
    - Yes
    - No
    - Sometimes
2. **During coronavirus pandemic, do you do any exercise (walking, running, equipment, etc.)?**
    - Never
    - 1-3 times/week
    - 4-5 times/week
    - Everyday
3. **Before coronavirus pandemic, did you use to do household chores?**
    - Never
    - 1-3 times/week
    - 4-5 times/week
    - Everyday
4. **During coronavirus pandemic, do you do household chores?**
    - Never
    - 1-3 times/week
    - 4-5 times/week
    - Everyday
5. **Before coronavirus pandemic, how much time did you spend on the computer for work/ study daily?**
    - None
    - 1-2 hours
    - 3-5 hours
    - More than 5 hours
6. **During coronavirus pandemic, how much time do you spend on the computer for work/ study daily?**
    - None
    - 1-2 hours
    - 3-5 hours
    - More than 5 hours

7. **Before coronavirus pandemic, how much time did you spend daily on Television, computer, social media for entertainment?**
    - Less than 30 minutes
    - 1-2 hours
    - 3-5 hours
    - More than 5 hours

8. **During coronavirus pandemic, how much time do you spend daily on Television, computer, social media for entertainment?**
    - Less than 30 minutes
    - 1-2 hours
    - 3-5 hours
    - More than 5 hours

Stress and Irritability

1. **How often have you experienced the following before coronavirus pandemic?**

	All the time	A large part of the time	Part of the time	A small part of the time	Not at all
Physically exhausted					
Emotionally exhausted					
Irritable					
Tense					

2. **How often have you experienced the following during coronavirus pandemic?**

	All the time	A large part of the time	Part of the time	A small part of the time	Not at all
Physically exhausted					
Emotionally exhausted					
Irritable					
Tense					

Sleep

1. **Before coronavirus pandemic, how many hours did you sleep per night?**
    - Less than 7 hours
    - 7-9 hours
    - More than 9 hours

2. **During coronavirus pandemic, how many hours are you sleeping per night?**
    - Less than 7 hours
    - 7-9 hours
    - More than 9 hours
3. **How would you rate your sleep quality before coronavirus pandemic?**
    - Very good
    - Good
    - Poor
4. **How would you rate your sleep quality during coronavirus pandemic?**
    - Very good
    - Good
    - Poor
5. **Before coronavirus pandemic, did you experience any of the following? (choose all that apply)**
    - Slept badly and restlessly
    - Hard to go to sleep
    - Woken up too early and not been able to get back to sleep
    - Woken up several times and found it difficult to get back to sleep
    - None of the above
6. **During coronavirus pandemic, do you experience any of the following? (choose all that apply)**
    - Slept badly and restlessly
    - Hard to go to sleep
    - Woken up too early and not been able to get back to sleep
    - Woken up several times and found it difficult to get back to sleep
    - None of the above
7. **How would you describe your energy level before coronavirus pandemic?**
    - Energized
    - Neutral
    - Lazy
8. **How would you describe your energy level during coronavirus pandemic?**
    - Energized
    - Neutral
    - Lazy
